# Investigating Home Range, Movement Pattern, and Habitat Selection of Bar-headed Geese during Breeding Season at Qinghai Lake, China

**DOI:** 10.3390/ani8100182

**Published:** 2018-10-18

**Authors:** Ruobing Zheng, Lacy M. Smith, Diann J. Prosser, John Y. Takekawa, Scott H. Newman, Jeffery D. Sullivan, Ze Luo, Baoping Yan

**Affiliations:** 1 University of Chinese Academy of Sciences, Beijing 100049, China; zhengruobing@cnic.cn; 2 e-Science Technology and Application Laboratory, Computer Network Information Center, Chinese Academy of Sciences, Beijing 100190, China; ybp@cnic.cn; 3 U.S. Geological Survey, Western Ecological Research Center, Vallejo, CA 94592, USA; lmsmith.sfbe@gmail.com; 4 U.S. Geological Survey, Patuxent Wildlife Research Center, Beltsville, MD 20705, USA; dprosser@usgs.gov; 5 Suisun Resource Conservation District, Suisun City, CA 94585, USA; jtakekawa@suisunrcd.org; 6 Food and Agriculture Organization of the United Nations, Regional Office for Africa, Accra, GA015, Ghana; Scott.Newman@fao.org; 7 Natural Systems Analysts, Winter Park, FL 32789, USA; jdsullivan@usgs.gov

**Keywords:** Bar-headed Goose, home range, movement pattern, habitat selection, breeding season, Qinghai Lake

## Abstract

**Simple Summary:**

The Bar-headed Goose is an important species in Asia, both culturally and ecologically. While prior studies have shown Qinghai Lake supports one of the largest breeding areas for Bar-headed Geese, little is known regarding the species movement ecology during the breeding season. In this study, we examined Bar-headed Goose home range size within the breeding grounds at Qinghai Lake and documented their daily movement patterns and habitat selection. We also identified several key breeding sites surrounding Qinghai Lake. Our research provides valuable information on this sensitive species that could help develop the strategy for waterfowl conservation and disease control.

**Abstract:**

The Bar-headed Goose is the only true goose species or Anserinae to migrate solely within the Central Asian Flyway, and thus, it is an ideal species for observing the effects of both land use and climate change throughout the flyway. In this paper, we investigate the home range, movement pattern, and habitat selection of Bar-headed Geese (*Anser indicus*) during the breeding season at Qinghai Lake, which is one of their largest breeding areas and a major migration staging area in the flyway. We identified several areas used by the geese during the breeding season along the shoreline of Qinghai Lake and found that most geese had more than one core use area and daily movements that provided insight into their breeding activity. We also observed the intensive use of specific wetlands and habitats near Qinghai Lake. These data provide interesting insights into the movement ecology of this important species and also provide critical information for managers seeking to understand and respond to conservation concerns threatening Bar-headed Geese, such as landscape and habitat changes.

## 1. Introduction

The Bar-headed Goose (*Anser indicus*) is a colonial nesting waterfowl species that breeds in Central Asia and winters as far south as the southern tip of India [1]. The Bar-headed Goose has notable cultural importance in China and Tibet, such as inspiring characters in ancient Sanskrit literature [2]. It is perhaps best known for its unique ability to migrate across the world’s highest mountains (e.g., the Himalayas) [3]. This migratory strategy has been of interest to researchers as they seek to understand the highly adaptive physiological and behavioral strategies that enable such a feat [4]. The migration of Bar-headed Geese to high elevation breeding ranges is fairly unique among Anserinae, as they are only one of the few species in this subfamily (others are the Canada Goose (*Branta canadensis*), Hawaiian Goose (*Branta sandvicensis*), and the Swan Goose (*Anser cygnoides*)) that do not migrate to breeding ranges at subarctic or Arctic latitudes [5].

In addition to their unique migratory behaviors and cultural significance, Bar-headed Geese present a unique opportunity to study how the changes within the Central Asian Flyway may affect waterfowl populations. Bar-headed Geese serve as ideal indicator species for this region as they are only one of the two species of true geese numbering in the thousands to winter on the Indian subcontinent [5], and previous telemetry work indicates that Bar-headed Geese migrate entirely within the Central Asian Flyway [6,7]. Bar-headed Geese also use some of this region’s most vulnerable habitat. For instance, the Qinghai-Tibetan Plateau, where a sub-population [4,8,9] totaling as much as 15% of the global Bar-headed Goose population breeds [10], has been found to be extremely prone to the impacts of climate change [5,11]. 

Bar-headed Geese may also provide the opportunity to examine how waterfowl in this region are affected by anthropogenic land use changes. While Bar-headed Goose populations have been feared to be in decline [1], a recent survey indicates that the global population may have increased to approximately 97,000–1,118,000 individuals as of 2014 [12]. It has been suggested that this recent population growth may be driven by the increased agricultural land use [12]. Thus, while enhanced population numbers are welcome news to conservationists, researchers, and the public alike, there are some concerns that agriculturally-supplemented populations may damage vital ecosystems such as those at Qinghai Lake, as has been seen with Snow Goose (*Chen caerulescens*) populations [13] in other locations. Thus, understanding how Bar-headed Geese utilize habitats on Qinghai-Tibetan Plateau may provide insights into the potential effects of rapidly increasing populations on their breeding grounds.

In a previous study, Cui et al. [14] reported that Bar-headed Geese utilized different portions of the Qinghai Lake complex to fit their shifting needs during pre-nesting, nesting, and moulting. However, questions remain regarding their preferred habitats and how individuals move at the daily level. This paucity of information constraints not only our understanding of basic species ecology but also the ability of researchers and managers to accurately identify the risks that these birds face and to properly prepare for and respond to future conservation risks. The objective of this study is to help fill the gaps in our understanding of Bar-headed Goose habitat use and movement patterns. We provide a novel examination of movements and space use at the daily level and investigate habitat preference throughout the breeding season. 

## 2. Study Area 

**Figure 1:**
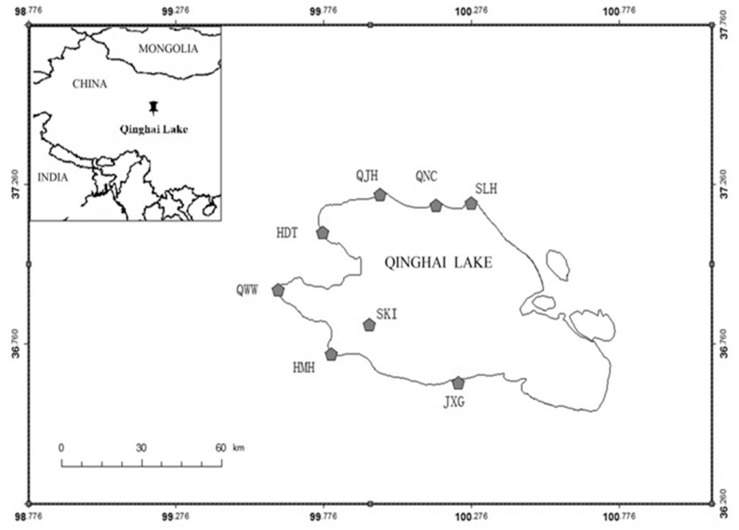
Map of Qinghai Lake in China 37.625° N, 98.772° E, 36.255° N, 101.011° E), where Bar-headed Geese (Anser indicus) were tracked by satellite telemetry during the breeding season. Wetland site codes are JXG (Jiangxigou), HMH (Heimahe Estuary), SKI (Sankuaishi Island), QWW (Quanwan), HDT (Hadatan), QJH (Quanjihe Estuary), QNC (Nongchang), and SLH (Shaliuhe Estuary).

This study took place at Qinghai Lake (37.817° N, 99.817° E) located west of Xining in Qinghai Province [15] on the Tibetan-Qinghai Plateau. Qinghai Lake is China’s largest saltwater lake with a surface area of 4317 km^2^ and an elevation of 3205 m (Figure 1). The lake is a closed basin fed by 25 freshwater streams with the intermittent flow [16], is frozen from November to March and has a short rainy season from June to August (0.35 m annual average rainfall) [17]. Located in the Central Asian Flyway, Qinghai Lake is an important wetland complex supporting many migratory water-bird species. The lake and surrounding wetlands serve as critical breeding habitat and a migratory staging area for more than 150,000 waterbirds each year [18]. Due to its ecological significance, Qinghai Lake has received multiple international designations including being recognized as a Key Staging Site for migrating Anatidae (waterfowl) [10] and a National Nature Reserve of China.

## 3. Methods

### 3.1. Capture and Marking

We captured and marked 29 Bar-headed Geese at three sites along Qinghai Lake: Jiangxigou (36.433° N, 100.167° E), Hadatan (37.067° N, 99.433° E), and Heimahe (36.433° N, 99.467° E) (Figure 1). We captured geese on 25–31 March 2007 and 28 March–3 April 2008 using monofilament leg nooses. We targeted equal numbers of male and female adult geese for marking. Each bird was equipped with a 45 g solar-powered portable transmitter terminal (PTT: Microwave Telemetry PTT-100, Columbia, Maryland, USA). We attached transmitters dorsally between the wings using Teflon harnesses (Bally Ribbon Mills, Bally, PA, USA). Transmitters weighed on average <2.1% of the goose’s body mass. We programmed transmitters to record GPS locations every two hours and data were uploaded to satellites every two days (CLS America Inc., Lanham MD, USA). Procedures for capture, handling, and marking were reviewed and approved by the Animal Care and Use Committees of the U.S. Geological Survey Patuxent Wildlife Research Center and the University of Maryland, Baltimore County (Protocol EE070200710). After the preliminary review of the data, we selected individuals with data spanning the study period and with sufficient daily data points to conduct our analyses.

### 3.2. Data Analysis

#### 3.2.1. Home Range and Movement Pattern

We used the Brownian Bridge Movement Model (BBMM) [19] to estimate the home ranges of marked Bar-headed Geese during the breeding season, which we defined as 1 April–1 July. The analysis was performed via the adehabitatHR package in R [20]. For birds that departed the Qinghai Lake region prior to 1 July, the analysis was ended at the time of their departure. Differing from classical kernel-based home range estimators, BBMM takes the time information between two successive relocations into account. It uses a kernel function for each step of the movement trajectory, where a step is a straight line connecting the two successive relocations. This kernel function combines two bivariate normal probability density functions and the Brownian Bridge probability density function [19,21]. BBMM deals with the issue of serial auto-correlation and unequal time intervals between locations in a straightforward manner by incorporating time into the model specifically instead of assuming independence between animal locations. For each goose, we created Brownian Bridge utilization distributions at 95% (home range) and 50% (core area) contour levels during the breeding season. We tested for differences in home range and core area size by sex with a Mann–Whitney U-test. 

We estimated daily home ranges of individuals during the breeding season with Keating’s kernel estimator [22] via the kernel kc function in R [20]. This method places a three-dimensional kernel function (X and Y coordinates and date) over each location and sums these kernel functions. Within each day, we use time as a linear variable and the bi-weight kernel function. We plotted the dynamic change of daily home range and used a Friedman test [23] to examine the differences in season-long daily home range sizes across individuals. For each individual, we also tested the difference in daily home ranges between the months using the Mann–Whitney U-test. To characterize the daily movements, we calculated movement distance and mean hourly movement rates per day. We estimated each bird’s daily movement distance by summing the Euclidean distance between all consecutive fixes within that day. The daily movement rate was calculated by dividing the Euclidean distance between consecutive fixes by the time interval separating those fixes. The mean of these values was then found for each calendar day. 

#### 3.2.2. Habitat Selection

We investigated habitat preference with the Manly selectivity measure [24], a widely-used approach to the study of habitat selection that defines several habitat categories on the study area and compares the use and availability of each habitat category. This method tests habitat selection with the log-likelihood ratios, and the selection ratio (used/available) for each habitat category is computed. More details of this methodology can be seen in Manly et al. [24]. 

To develop habitat maps to estimate habitat selection, we obtained and resampled multi-source remote sensing data to provide different habitat characteristics and we used ESA GlobCover 2009 to determine the land cover type. We excluded the large deep-water region with a 2-km buffer around the lakeshore. We created a Normalized Difference Vegetation Index (NDVI) map from MODIS MOD13Q1 and extracted the water body from Landsat TM (LS5 TM 20070518) based on the Normalized Differential Water Index (NDWI) and manually corrected any areas falsely categorized as water within the study area. We determined elevation with the study area and created two distance maps by computing the Euclidean distance between each pixel to the water body as well as major roads. We processed data and conducted analyses using Python 2.7.3, Geospatial Data Abstraction Library (python-GDAL 1.7.3), SciPy (Open Source Library of Scientific Tools), R-packages (adehabitatHR, adehabitatHS), ArcMap 10.0 (ESRI, Inc., Redlands, CA, USA), and ENVI 5.0 (Exelis VIS).

## 4. Results

### 4.1. Marking and Telemetry

Of the 29 birds marked, we used eight Bar-headed Geese with comprehensive breeding season datasets: four (two males and two females) marked in 2007, and four (two males and two females) marked in 2008. We continuously monitored these eight geese within the study area during the breeding season and obtained 2876 GPS relocations, ranging from 234 to 573 locations per goose (Table 1).

### 4.2. Home Range and Movement Pattern

**Figure 2:**
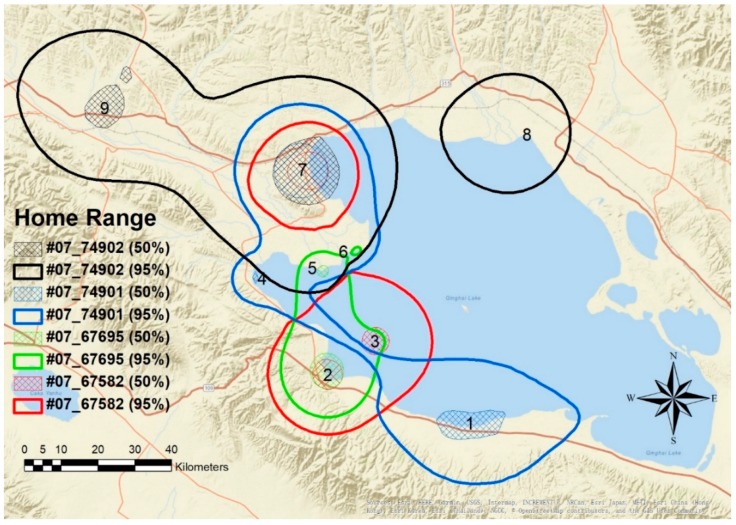
Home range (95% contour) and core area (50% contour) of four Bar-headed Geese (*Anser indicus*) captured in 2007 in Qinghai Lake (March-October) created using a Brownian Bridge Movement Model. Core areas and codes: 1. Jiangxigou, 2. Heimahe Estuary, 3. Sankuaishi Island, 4. Garila and Quanwan, 5. Shenhe Estuary, 6. Buhahe Estuary and Egg Island, 7. Hadatan, 8. Shaliuhe Estuary, and 9. Luonia.

**Figure 3:**
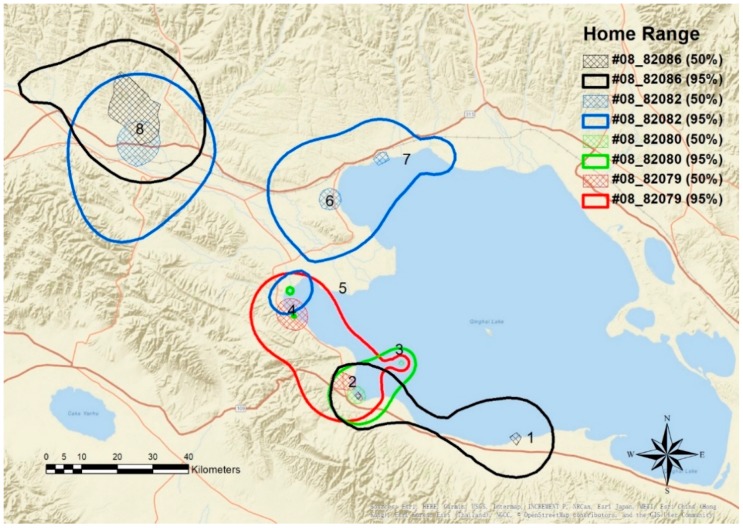
Home range (95% contour) and core area (50% contour) of four Bar-headed Geese (*Anser indicus*) captured in 2008 in Qinghai Lake (March-September) created using a Brownian Bridge Movement Model. Core areas and codes: 1. Jiangxigou, 2. Heimahe Estuary, 3. Sankuaishi Island, 4. Garila and Quanwan, 5. Shenhe Estuary, 6. Hadatan, 7. Quanjihe Estuary, and 8. Luonia.

Most geese used the estuary and wetlands around Qinghai Lake heavily (Figure 2 and Figure 3). Sites used during the breeding period were scattered over the west and north shoreline of Qinghai Lake, including Heimahe Estuary, Hadatan, Garila and Quanwan wetland, Luonia, Jiangxigou, Sankuaishi Island, Shenhe Estuary, Buhahe Estuary, Egg Island, QuanjiHe Estuary, Shaliuhe Estuary, Cuolongka, and Cuonariama. There was a high degree of home range and core use area overlap between the individuals, and all geese had more than one core use area located among the different sites.

Overall home ranges averaged 1415 km^2^ and core areas averaged 113 km^2^ (Table 2). Interestingly, female geese had larger home ranges (2680 ± 1108 km^2^; W = 16, *p* = 0.014) than male geese. There was no significant difference in the size of core areas (W = 13, *p* = 0.1) between sexes. There was also no significant difference in overall home-range (W = 10, *p* = 0.34) or core area size between years (W = 13, *p* = 0.1). In general, geese moved a mean distance of 6.02 km per day while averaging 0.76 km/h (Table 3).

**Figure 4:**
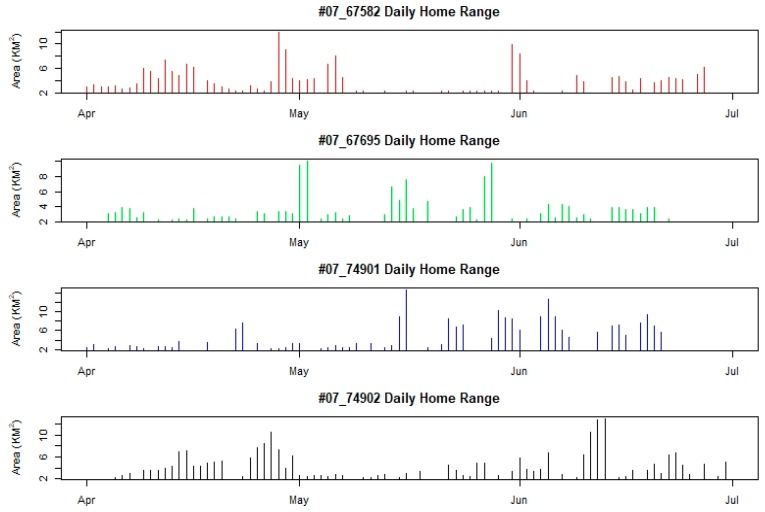
The dynamic change of daily home range sizes (km^2^) of four Bar-headed Geese (*Anser indicus*) marked and satellite-telemetry tracked at Qinghai Lake in 2007. Birds 07_67582 and 07_74902 are female while 07_67695 and 07_74901 are male.

**Figure 5:**
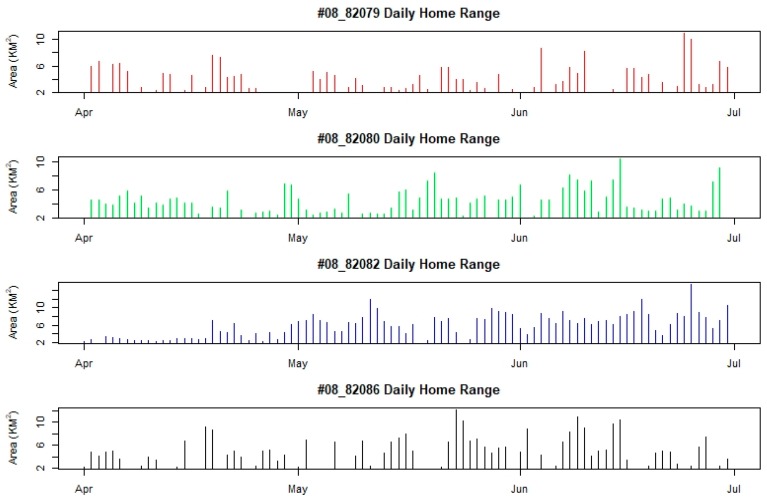
The dynamic change of daily home range sizes (km^2^) of four Bar-headed Geese (*Anser indicus*) marked and satellite-telemetry tracked at Qinghai Lake in 2008. Birds 08_82082 and 08_82086 are female while 08_82079 and 08_82080 are male.

We observed no significant difference in daily home ranges between individuals in 2007 (Friedman *χ*^2^ = 3.2667, df = 3, *p*-value = 0.3523) or 2008 (Friedman *χ*^2^ = 4.7333, df = 3, *p*-value = 0.1924). However, within a given year, individuals displayed dramatically different space use patterns between months (Figure 4 and Figure 5). For example, #67582 had a daily home range in April and June that was significantly larger than its daily home range in May (W = 417.5, *p*-value = 0.0045; W = 263.5, *p*-value = 0.0064). In contrast, #67695 had a daily home range in April that was significantly smaller than its home range in May (W = 129, *p*-value = 0.0178) and a daily home range in May significantly smaller than its home range in June (W = 57, *p*-value = 0.0075). 

### 4.3. Habitat Selection

Our land cover preference analysis showed that Bar-headed Geese preferred wetland and open land with sparse vegetation significantly more than other habitats (${\chi_{L}}^{2}$ = 2700.529, df = 4, *p* < 0.001), and that they avoided forest and croplands (Table 4). Similarly, NDVI distribution preferences indicated that geese preferred wetland and sand areas to dense vegetation (${\chi_{L}}^{2}$ = 324.91, df = 3, *p* < 0.001). All geese showed a preference for sites close to rivers and the lake (${\chi_{L}}^{2}$ = 2751.1, df = 3, *p* < 0.001), and they primarily occupied elevations of 2.8–3.3 km (${\chi_{L}}^{2}$ = 2167.94, df = 4, *p* < 0.001) and avoided higher elevations that are widely distributed throughout the study area. Even though the main breeding sites within the Qinghai Lake area were surrounded by several highways and railways, Bar-headed Geese still showed a preference for keeping greater than 0.5 km away from roads (${\chi_{L}}^{2}$ = 1260.51, df = 3, *p* < 0.001).

## 5. Discussion

In this study, we used telemetry data from Bar-headed Geese to gain insight into the movement ecology of this species during the breeding season at Qinghai Lake. To meet this objective, we analyzed home range size daily and seasonally, as well as the daily movement patterns and habitat selection. This study represents a comprehensive examination of the spatial and temporal dynamics of highly mobile birds during the breeding season at a critical but vulnerable location.

### 5.1. Home Range and Movement Pattern

A primary finding of this study was documenting the variability in daily home ranges between months for individual birds. We believe that variation in their movement was likely driven by different breeding activity and examining daily movement patterns provided insight into the breeding status of individuals. For instance, in 2007 males had relatively low daily movement rates from April until early May, which coincided with the pre-nesting period. Such behavior would likely indicate that males were establishing territories or nesting sites during this time [25]. Similarly, females appeared to restrict daily movements from mid May–early June during the breeding and nesting period which indicated their greatly reduced movements while they incubated eggs and began rearing chicks. Male movements were less constricted during this time as they do not aid in incubation, although they do remain vigilant near the nest and assist in raising chicks [26]. Both sexes increased movement rates around the time when chicks would become mobile and adults could move their brood to other core areas. In contrast, females in 2008 did not display daily movement patterns that would indicate breeding activity, despite both individuals remaining within a single breeding area throughout the nesting period. Such behavior would suggest that these females did not try to reproduce or were not successful. Both years had relatively comparable precipitation and weather conditions [27].

The use of multiple core areas during the breeding season suggested that space use was driven by differing resource needs. For instance, while birds would breed in one area, they would travel to a separate location later in the season, likely to access different food resources for young chicks and females recovering from the nutritional stress of reproduction, which is typical for waterfowl. The search for food stocks sufficient for females to recover from the demands of reproduction may also explain why females had larger seasonal home ranges than males. Generally, geese tended to use the same core area within a given day, resulting in shorter daily movement distances. Larger daily movement distances and movement rates corresponded to shifts between different core areas. Thus, geese were not moving between the core areas regularly but instead would remain in one area for extended periods of time. This means that much of the Qinghai Lake complex was important for Bar-headed Geese at different times of the breeding period across different habitats.

Our approach provides novel insight into both daily and seasonal space use of Bar-headed Geese. Seasonal home range values reported in this study vary greatly from those reported in Cui et al [14], but they divided the breeding period into three sub-periods and developed kernel home ranges which do not take the temporal structure of the data into consideration like Brownian Bridge Movement Models [28]. Although movements of monitored individuals may have been influenced by tracking devices [29,30], localized movements such as those reported in this study may be less impacted. While only a few individuals at Qinghai Lake were monitored, we observed no results which would suggest that these results are not representative of the broader population.

### 5.2. Habitat Selection

Bar-headed Geese used the area near rivers and the lake more than expected. They selected wetlands and lakefront, showing a significant preference for wet habitats, and generally avoided croplands. The avoidance of croplands was unexpected, as this species has regularly been observed in grain fields during the non-breeding season [31]. However, this behavior suggests that wild food stocks within their core use areas were sufficient to limit the use of agricultural areas. It also indicates that while enhanced crop availability may be driving increases in Bar-headed Goose populations on wintering grounds [12], increasing agricultural land use may not change their distribution while on the breeding grounds. It indicates that the breeding area may not be able to support dramatically increased populations should Bar-headed Geese numbers expand rapidly as a result of increased access to agricultural food stocks on the wintering grounds.

### 5.3. Conservation Implications

We found that Bar-headed Geese used several habitat types on their breeding grounds. They selected natural wetland habitats over agricultural croplands, and those areas may be of key importance for their productivity. Furthermore, the use of multiple core areas by birds indicated that preserving only a few selected locations of one habitat type will be insufficient to protect the population. Instead, efforts must be made to protect the diverse assemblage of habitat types across the multiple locations at Qinghai Lake. Such conservation measures may be difficult in the face of rapid development along the shores of Qinghai Lake [32], but it is essential to ensure a stable breeding population at this key site.

One major future conservation concern for the Bar-headed Geese is the impact of climate change on habitat quality and availability. Climate-change related influences on the Tibetan-Qinghai Plateau wetland habitats are predicted to be particularly dramatic [17,33]. Annual temperatures have increased in parallel with warming over the Northern Hemisphere, and the rate of increase is positively correlated with elevation [11,34]. In northern regions, warming trends have led to arid conditions and lowered rainfall, causing drought and reduced vegetation growth. The southern region has experienced the opposite effect, including more humid weather and increased vegetation growth [35]. Change in habitat composition in the Qinghai Lake region may force geese to increase their use of agricultural areas as natural vegetation declines. A decrease in preferred habitat may also increase overall water-bird density, thereby increasing disease transmission risk and competition. 

An additional challenge to Bar-headed Goose subpopulations that breeds at Qinghai Lake is the threat of avian influenza. The overlap of bird’s core use areas indicates a greater potential for direct interaction which could result in disease transmission. This transmission potential is especially concerning for Bar-headed Geese, as this species is particularly susceptible to highly pathogenic avian influenza H5N1 [36]. Prosser et al. [6] suggested that Bar-headed Geese could transport avian influenza viruses to Qinghai Lake from their wintering grounds either directly or via other birds encountered during stopovers. Furthermore, wetlands surrounding Qinghai Lake are important feeding areas for many species of water birds, including Great Black-headed Gull (*Ichthyaetus ichthyaetus*), Brown-headed Gull (*Chroicocephalus brunnicephalus*), Ruddy Shelduck (*Tadorna ferruginea*), Great Cormorant (*Phalacrocorax carbo*), and other species that may potentially transmit H5N1 [14]. Interactions between species and individuals could increase the risk of virus transmission. Thus, the improved understanding of core use areas and movement patterns provided through this study could be used to help guide the monitoring of disease outbreaks. Future work should consider making comparisons of core areas and movement patterns between Bar-headed Geese and other species to highlight species overlap and disease transmission risk at this important breeding grounds. 

## 6. Conclusions

We investigated the home range for multiple Bar-headed Geese during the breeding season at Qinghai Lake and provide new insights into local movement pattern. We documented the existence of multiple Bar-headed Geese breeding sites at Qinghai Lake and made a comprehensive comparison of home range and local movement pattern from different perspectives. Additionally, we identified habitat characteristics selected by geese within the breeding ground. Our work offers detailed information of Bar-headed Goose space use that will be required to address a variety of future challenges facing this species. The results of this study will be helpful to the development of conservation strategies and could help guide monitoring of disease outbreaks.

## Figures and Tables

**Table 1:** ID number, sex, capture and departure dates, number of satellite GPS locations, and home range and core area size for Bar-headed Geese (*Anser indicus*) marked and satellite-tracked at Qinghai Lake.

ID	Sex	Capture Date	Departure Date	Number of GPS Fixes	Home Range Area (km^2^)	Core Area (km^2^)
07_67582	F	03/25/07	07/02/07	327	1476.15	126.5
07_67695	M	03/29/07	06/22/07	252	543.89	49.22
07_74901	M	03/31/07	06/21/07	234	1369.48	187.62
07_74902	F	03/30/07	10/24/07	334	3910.36	242.03
08_82079	M	04/02/08	08/31/08	329	612.82	58.42
08_82080	M	04/02/08	06/29/08	561	211.57	13.57
08_82082	F	03/30/08	09/29/08	573	1788.28	103.63
08_82086	F	03/31/08	08/21/08	266	1409.34	120.37

**Table 2:** Mean (±SD) home range (95% contour) and core area (50% contour) size of Bar-headed Geese (Anser indicus) marked and tracked at Qinghai Lake, during breeding seasons of 2007 and 2008.

Year	n	Home Range (km^2^) (min–max)	Core Area (km^2^) (min–max)
Female	4	2146.03 ± 1187.76 (1409.34–3910.36)	245.34 ± 89.41 (137.84.86–321.61)
Male	4	990.14 ± 778.22 (220.36–2011.66)	84.93 ± 75.21 (16.23–191.49)
2007	4	1987.93 ± 1419.39 (543.89–3910.36)	148.13 ± 63.34 (103.63–242.03)
2008	4	684.44 ± 489.14 (211.57–1369.48)	77.21 ± 76.11 (13.57–187.62)

**Table 3:** Mean (±SD) daily home range, mean (±SD) and median daily movement distance, and mean (±SD) and median daily movement rate of Bar-headed Geese (*Anser indicus*) captured in 2007 and 2008 at Qinghai Lake.

ID	Sex	Daily Home Range (km^2^) (min–max)	Daily Movement Distance (km) (min–max)-Median	Daily Movement Rate (Km/H) (Min–Max)-Median
07_67582	F	4.08 ± 2.03 (2.30–11.95)	4.07 ± 13.34 (0–96.23)-0.87	0.31 ± 0.78 (0–4.37)-0.09
07_67695	M	3.71 ± 1.84 (2.30–10.05)	3.24 ± 7.42 (0–40.22)-0.71	0.25 ± 0.49 (0–2.51)-0.08
07_74901	M	5.04 ± 2.99 (2.20–14.62)	5.34 ± 11.81 (0–73.70)-0.94	2.52 ± 0.94 (0–4.82)-0.13
07_74902	F	4.42±2.43 (2.25–12.99)	4.27 ± 9.03 (0–51.67)-1.34	0.39 ± 0.99 (0–7.24)-0.12
08_82079	M	4.64 ± 1.95 (2.30–11.00)	4.64 ± 7.92 (0–35.56)-1.88	0.51 ± 1.08 (0–6.98)-0.18
08_82080	M	4.52 ± 1.73 (2.32–10.49)	5.89 ± 8.43 (0–37.32)-1.83	0.52 ± 0.86 (0–3.19)-0.12
08_82082	F	5.96 ± 2.68 (2.31–15.40)	12.76 ± 23.58 (0–195.49)-4.28	0.87 ± 1.51 (0–12.22)-0.24
08_82086	F	5.37 ± 2.44 (2.17–12.24)	7.99 ± 12.53 (0–78.34)-3.78	0.74 ± 1.12 (0–6.00)-0.33

**Table 4:** The result of Manly’s habitat selection ratios for Bar-headed Geese (Anser indicus) captured at Qinghai Lake in 2007 and 2008. The selection ratio ${\hat{w}}_{i}$ for each category *i* is calculated as ${\hat{w}}_{i}$ = used_*i*_/avail_*i*_.

Characteristic	Category					Bonferroni CI		Selection
		${\mathrm{used}}_{i}$	${\mathrm{avail}}_{i}$	${\hat{w}}_{i}$	$s.\;e({\hat{w}}_{i})$	Lower	Upper	
Land cover	Cropland	0.076	0.460	0.165	0.009	0.140	0.190	-
	Forest	0.207	0.302	0.685	0.026	0.615	0.754	-
	Shrubland	0.006	0.000	26.28	26.75	-44.31	96.87	0
	Barrenland	0.177	0.132	1.336	0.070	1.153	1.520	+
	Wetland	0.533	0.105	5.075	0.243	4.434	5.716	+
${\chi_{L}}^{2}$ = 2700.529, df = 4, *p* < 0.001								
NDVI	−1–−0.5	0.0001	0.0001	1.000	1.000	–1.576	3.576	0
	−0.5–0	0.339	0.185	1.836	0.071	1.652	2.020	+
	0–0.5	0.660	0.815	0.810	0.011	0.783	0.837	-
	0.5–1	0.000	0.000	1.000	1.414	–2.643	4.643	0
${\chi_{L}}^{2}$ = 324.91, df = 3, *p* < 0.001								
Distance to water (km)	0–0.5	0.796	0.309	2.578	0.063	2.421	2.735	+
	0.5–2	0.125	0.241	0.518	0.025	0.454	0.581	-
	2–4	0.076	0.178	0.428	0.027	0.361	0.496	-
	>4	0.003	0.272	0.010	0.003	0.002	0.017	-
${\chi_{L}}^{2}$ = 2751.1, df = 3, *p* < 0.001								
Distance to road (km)	0–0.2	0.013	0.029	0.444	0.071	0.266	0.621	-
	0.2–0.5	0.022	0.030	0.740	0.099	0.492	0.988	-
	0.5–2	0.531	0.169	3.138	0.116	2.847	3.428	+
	>2	0.434	0.771	0.563	0.011	0.535	0.590	-
${\chi_{L}}^{2}$ = 1260.51, df = 3, *p* < 0.001								
Elevation (km)	2.8–3.2	0.544	0.214	2.538	0.085	2.319	2.758	+
	3.2–3.3	0.289	0.255	1.130	0.041	1.025	1.236	+
	3.3–3.4	0.146	0.176	0.828	0.042	0.720	0.936	-
	3.4–3.6	0.021	0.184	0.112	0.012	0.080	0.144	-
	>3.6	0.001	0.170	0.006	0.003	-0.002	0.013	-
${\chi_{L}}^{2}$ = 2167.94, df = 4, *p* < 0.001								

+ stands for use significantly more than expected; - means use significantly less than expected; 0 means use in proportion to habitat availability.
